# Remifentanil target-controlled infusion for fiberoptic bronchoscopy in the ICU: feasability, safety and tolerance evaluation

**DOI:** 10.1186/2197-425X-3-S1-A325

**Published:** 2015-10-01

**Authors:** M Caron, A Parrot, C Blayau, EM Hafiani, JP Fulgencio, V Labbé, M Djibré, M Decavèle, C Quesnel, M Fartoukh, T Pham

**Affiliations:** Hôpital Tenon, Assistance Publique des Hôpitaux de Paris, Medical and Surgical ICU, Paris, France; Université Pierre et Marie Curie, Paris VI, Paris, France; Paris Descartes University, UMR 1153, Inserm, Sorbonne Paris Cité, ECSTRA, Paris, France

## Introduction

Remifentanil target-controlled infusion (TCI), widely used in the operating room owing to its reliability, is poorly assessed in the ICU, while several procedures may induce pain and discomfort. Only one series of 14 patients focused on Remifentanil-TCI use for performing fiberoptic bronchoscopy (FB) in the ICU [1]. We aimed to assess Remifentanil-TCI feasibility, safety and tolerance during FB performed in consecutive non-intubated ICU patients.

## Objectives

Primary end point was FB complete achievement, with Remifentanil TCI and standardized local anesthesia without need for general anaesthesia or additional analgesia. Secondary aims were objective (respiratory and hemodynamics parameters) and subjective (pain, dyspnea and comfort assessment) tolerance and safety measurements during and after the procedure.

## Methods

Observational prospective study of Remifentanil target-controlled infusion (TCI) use during FB performed in non-intubated ICU patients. Quantitative and qualitative variables are presented as median [interquartile range] and number (percentage), respectively.

## Results

From November 2014 to March 2015, 40 patients (age 59 yrs [41-71], 75% men; SAPS II score 28 [22-39]) were included. FB was performed in patients receiving nasal oxygen (85%), high-flow humidified oxygen (12.5%) or non-invasive ventilation (2.5%), and a broncho-alveolar lavage was done in 7 patients (17.5%). Total infusion duration was 22 minutes [15-29], TCI concentration goal was 4 ng/mL [3.5-5.5] and total drug dose infused wad 279 µg [196-377].

FB was performed completely without any additional drugs in 38 patients (95%), whereas propofol was used in 2 (5%). Three patients presented side effects comprising bradypnea (n = 1) and hypotension (systolic arterial pressure of 70 mmHg, n = 2) that occurred during the procedure and were completely reversible with TCI goal decrease. Thus, FB was performed entirely without additional anesthesia or complication in 35 patients (87.5%).

Comfort was assessed in 28 patients (70%): 22 (79%) stated that they experienced a bearable pain or no pain at all, 6 (21%) had a moderate pain, and none felt severe pain. Among the 26 patients who were asked, 20 (77%) would agree to have a FB in the same conditions.

## Conclusions

Our findings suggest that TCI of Remifentanil may be useful and safe, for performing FB in the ICU, with acceptable patients' comfort and tolerance, even in those with respiratory failure. The study completion should strengthen these preliminary results.
